# Improving acute myocardial infarction care in northern Tanzania: barrier identification and implementation strategy mapping

**DOI:** 10.1186/s12913-024-10831-5

**Published:** 2024-03-28

**Authors:** Julian T Hertz, Francis M Sakita, Sainikitha Prattipati, Lauren Coaxum, Tumsifu G Tarimo, Godfrey L Kweka, Jerome J Mlangi, Kristen Stark, Nathan M Thielman, Hayden B Bosworth, Janet P Bettger

**Affiliations:** 1 Duke Global Health Institute, Duke University School of Medicine, Durham, NC USA; 2 Department of Emergency Medicine, Duke University School of Medicine, Durham, NC USA; 3 Department of Emergency Medicine, Kilimanjaro Christian Medical Center, Moshi, Tanzania; 4 Kilimanjaro Christian Research Institute, Moshi, Tanzania; 5 Department of Internal Medicine, Duke University School of Medicine, Durham, NC USA; 6 Department of Population Health Sciences, Duke University, Durham, NC USA

**Keywords:** CFIR-ERIC, Acute myocardial infarction, Tanzania, Strategy mapping

## Abstract

**Background:**

Evidence-based care for acute myocardial infarction (AMI) reduces morbidity and mortality. Prior studies in Tanzania identified substantial gaps in the uptake of evidence-based AMI care. Implementation science has been used to improve uptake of evidence-based AMI care in high-income settings, but interventions to improve quality of AMI care have not been studied in sub-Saharan Africa.

**Methods:**

Purposive sampling was used to recruit participants from key stakeholder groups (patients, providers, and healthcare administrators) in northern Tanzania. Semi-structured in-depth interviews were conducted using a guide informed by the Consolidated Framework for Implementation Research (CFIR). Interview transcripts were coded to identify barriers to AMI care, using the 39 CFIR constructs. Barriers relevant to emergency department (ED) AMI care were retained, and the Expert Recommendations for Implementing Change (ERIC) tool was used to match barriers with Level 1 recommendations for targeted implementation strategies.

**Results:**

Thirty key stakeholders, including 10 patients, 10 providers, and 10 healthcare administrators were enrolled. Thematic analysis identified 11 barriers to ED-based AMI care: complexity of AMI care, cost of high-quality AMI care, local hospital culture, insufficient diagnostic and therapeutic resources, inadequate provider training, limited patient knowledge of AMI, need for formal implementation leaders, need for dedicated champions, failure to provide high-quality care, poor provider-patient communication, and inefficient ED systems. Seven of these barriers had 5 strong ERIC recommendations: access new funding, identify and prepare champions, conduct educational meetings, develop educational materials, and distribute educational materials.

**Conclusions:**

Multiple barriers across several domains limit the uptake of evidence-based AMI care in northern Tanzania. The CFIR-ERIC mapping approach identified several targeted implementation strategies for addressing these barriers. A multi-component intervention is planned to improve uptake of evidence-based AMI care in Tanzania.

**Supplementary Information:**

The online version contains supplementary material available at 10.1186/s12913-024-10831-5.

## Background

Acute myocardial infarction (AMI) is a life-threatening emergency associated with high morbidity and mortality [1, 2]. Over the past several decades, advances in evidence-based AMI care, including early diagnosis, rapid reperfusion strategies, antiplatelet therapy, and others, have led to significant improvement in morbidity and mortality [2–4]. Because optimal AMI care requires both early identification of AMI cases and rapid initiation of time-sensitive treatments, numerous studies have been conducted in resource-replete settings to improve AMI care in the emergency department (ED) [5–8]. In particular, studies have shown that AMI care can be improved by leveraging implementation science to support uptake of evidence-based interventions using strategies such as audit/feedback, checklists, and reminders to improve a wide range of process outcomes [5–8]. However, a recent systematic review of quality improvement strategies to improve AMI care found no such studies in low-income settings, including all of sub-Saharan Africa (SSA) [5]. 

Emerging evidence suggests that the incidence of AMI in SSA may be greater than previously believed and growing [9–12]. As the region advances through the epidemiologic transition and the burden of age-related non-communicable disease continues to rise, [13] increased attention will need to be directed towards providing timely, high-quality AMI care in EDs in SSA. Presently, little is known about current patterns of AMI care in SSA, with only a handful of published studies reporting quality metrics and even fewer studies examining ED-based care [11, 14, 15]. In Tanzania, we conducted a prospective study of AMI care in the ED of a tertiary care center and identified multiple gaps in evidence-based care: only half of adults presenting with acute chest pain underwent electrocardiogram (ECG) testing; cardiac biomarkers were obtained for less than 3% of patients with chest pain; approximately 90% of AMI cases were misdiagnosed; only 23% of AMI patients received aspirin in the ED; and 43% of patients with AMI died within 30 days of presentation [12, 16, 17]. These findings draw attention to the urgent need for a quality improvement intervention to increase uptake of evidence-based AMI care in Tanzania; unfortunately, proven strategies for improving AMI care in SSA are currently lacking [5, 18]. 

Identifying and intervening upon the reasons for poor uptake of these evidence-based therapies requires an approach rooted in implementation science. There is ample evidence to support national and international guidelines for AMI care; [3, 4, 19] the challenge in our setting is in implementing these guidelines. In order to inform the development of a locally-tailored approach to improve AMI care, we conducted a qualitative study of barriers to AMI care. Using the Consolidated Framework for Implementation Research (CFIR) [20] to guide data collection, we coded and categorized barriers to care into areas that could be addressed. We then applied the Expert Recommendations for Implementing Change (ERIC) tool [21] to map barriers to implementation strategies that could be employed to improve guideline uptake. Given the current dearth of implementation research addressing AMI care in SSA, our findings may inform those working to improve AMI care elsewhere in SSA or other resource-limited settings, where barriers and contextual factors may be similar.

## Methods

### Setting

This study was conducted in the Kilimanjaro Region of northern Tanzania, the site of our preliminary studies that identified gaps in evidence-based AMI care [12, 16, 17, 22]. The total population of the Kilimanjaro Region is approximately 1.9 million persons and the local prevalence of hypertension and diabetes among the adult population is approximately 27% and 6%, respectively [23–25]. The predominant local language is Swahili, although English is often used among healthcare providers in hospital settings. Inpatient and ED care are available to everyone at KCMC, regardless of insurance coverage. Most patients diagnosed with AMI in the KCMC ED are admitted to the KCMC inpatient medical ward, where they continue to receive AMI care such as anticoagulation and echocardiography. In some cases, patients with AMI are transferred directly to the nearest hospital capable of performing percutaneous coronary intervention, Jakaya Kikwete Cardiac Institute, which is approximately 10 h away by car. Approximately half of AMI patients admitted to KCMC have healthcare insurance coverage, the vast majority of whom have health insurance through government-funded insurance programs [12, 26]. Most elements of ED and inpatient AMI care are covered by Tanzanian health insurance plans; uninsured patients pay for their care out-of-pocket.

### Participant selection

We identified three key stakeholder groups whose perspectives are essential for discerning barriers to implementing evidence-based AMI care: patients, providers, and healthcare administrators. Patient participants were recruited from an existing cohort of patients with confirmed AMI enrolled in a recently completed surveillance study conducted in an ED in Kilimanjaro [12, 17, 22]. During enrollment in the original AMI surveillance study, patients had previously provided basic demographic information including gender, age, health insurance coverage, educational level, and household income. Age and socioeconomic characteristics guided purposive sampling of patients for this study to achieve a diversity of participants. Provider participants included physicians, clinical officers, and nurses working in EDs within the Kilimanjaro Region. A clinical officer is a mid-level advanced practice provider who studies clinical medicine for three years after secondary school and practices independently. Healthcare administrator participants included department heads, hospital directors, and charge nurses. Purposive sampling was used to recruit providers and healthcare administrators from these roles across a range of hospitals in Kilimanjaro. Although providers and administrators were recruited from multiple hospitals, only patients who had been diagnosed with AMI at KCMC were invited to participate.

### In-depth interviews

A semi-structured in-depth interview guide was developed by an interdisciplinary team of implementation scientists, social scientists, and physicians. The interview guide was designed using CFIR, which provides a structured approach to assess facilitators and barriers to implementation across five domains [20]. The interview guides, developed specifically for this study, are included in the supplementary material (see Supplementary Material). The research assistants conducting in-depth interviews had prior experience performing in-depth interviews, [26, 27] and they underwent additional training led by two US-based implementation scientists (JTH and JPB) in using the semi-structured interview guide. Informed consent was obtained prior to the interviews, and participants were reimbursed 5,000 Tanzanian shillings (approximately 2 USD) for their time. Interviews were conducted face-to-face in either Swahili or English (whichever the participant preferred), in a private room, for approximately 1 h. Interviews were audio-recorded, transcribed, and translated into English. An investigator fluent in both Swahili and English (JTH) reviewed the transcripts and audio recordings to ensure accuracy. Interviews were conducted over a six-month period, from October 2021 through April 2022.

### Qualitative analysis

Prior to starting qualitative analysis, all members of the coding team underwent training in the CFIR coding guide which outlines 39 constructs across five domains [20]. For purposes of our thematic analysis, we defined the five CFIR domains thusly: “Innovation” referred to evidence-based AMI care or strategies to improve uptake of such care; “Inner Setting” referred to the hospital, healthcare providers, and the healthcare setting; “Outer Setting” referred to all settings outside of the hospital; “Individuals” referred to patients; and “Process” referred to process of providing AMI care or improving such care. All transcripts were independently double-coded by two members of the coding team (JTH, JPB, TGT, GLK, JJM, LC, SP, manually, without software). Coders identified any barrier or facilitator to AMI care revealed in interview responses and coded it according to the relevant CFIR construct. Coding disagreements were resolved by consensus, and iterative refinements to the codebook were made throughout the coding process to reflect decisions on best fit for experiences and perspectives that fit multiple constructs (or none at all). The final codebook is provided as supplementary material.

Based on our prior experience conducting in-depth interviews in Tanzania, [26, 27] the investigator team decided *a priori* to conduct 10 in-depth interviews with members of each stakeholder group and then conduct an interim thematic analysis to determine whether or not thematic saturation had been achieved prior to proceeding with additional interviews. Coded transcripts for ten stakeholders from each stakeholder group (patients, providers, and administrators) were initially interviewed and thematic analysis of these interview transcripts was conducted as described above. Two members of the coding team then reviewed each set of transcripts to determine whether thematic saturation had been achieved. Thematic saturation was defined as no new facilitator or barrier identified in the 9th and 10th interview for each stakeholder group. In each case, the coders determined that thematic saturation had been achieved; consequently, no additional interviews were conducted.

Codes were then analyzed to identify intervenable barriers. Within each of the 39 CFIR constructs, a pair of coders (JTH and JPB) conducted rapid thematic analysis to identify the specific barrier or facilitator referenced. For example, within the CFIR domain of “Outer Setting” and the construct “External Policies and Incentives,” the team further coded relevant sub-constructs, such as insurance coverage, government subsidies, and referral systems. The pair of team members then reviewed the full list of codes to identify the most common barriers relevant to ED-based AMI care (as opposed to preventative care, subacute care, or community-based care, for example). Additional facilitators and barriers identified during thematic analysis that were relevant to other phases of AMI care or that were not intervenable in an ED-based intervention (such as health insurance coverage policies) will be the subject of a separate analysis.

**Fig. 1:**
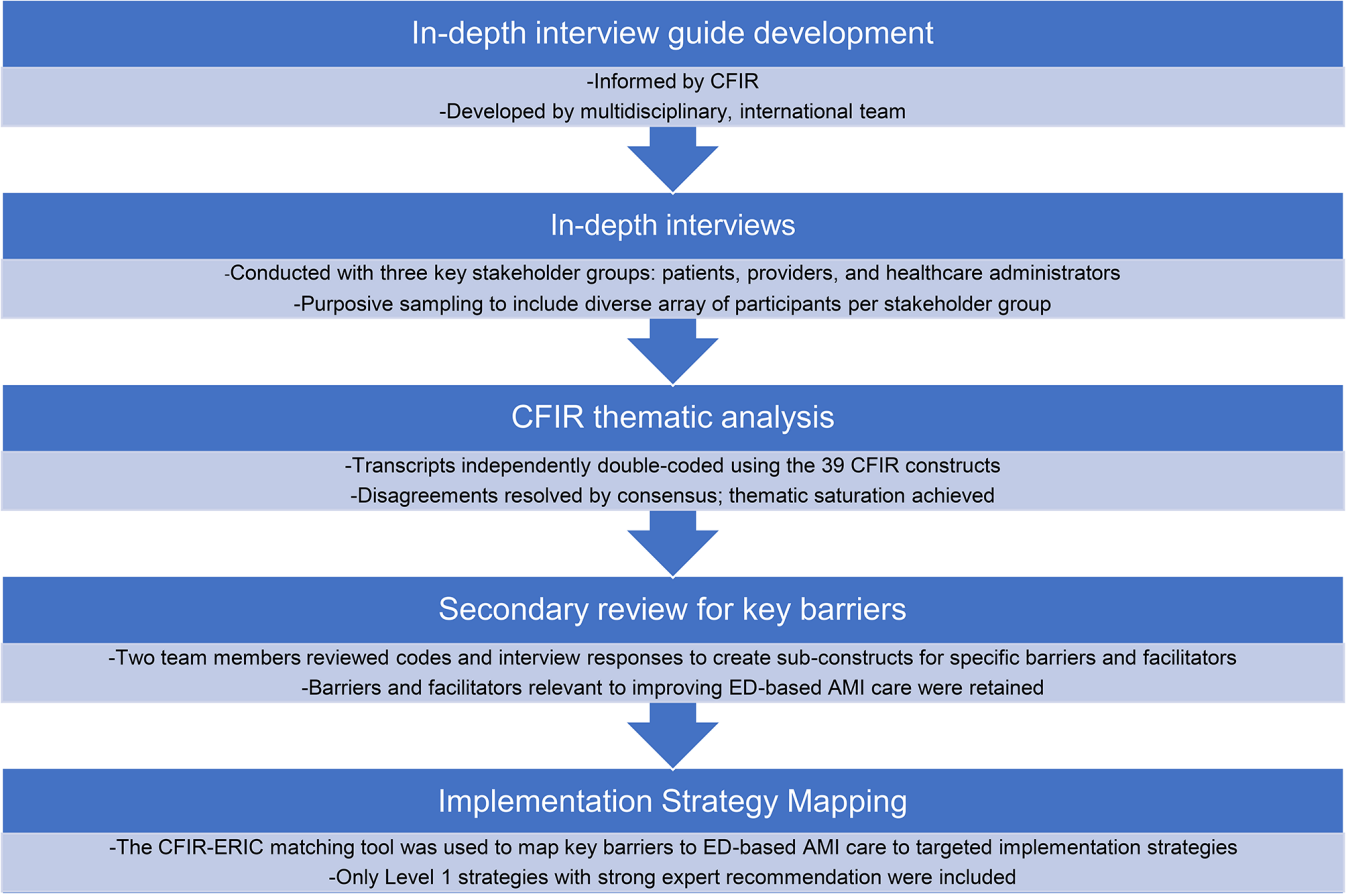
Summary of analytic approach for identifying key barriers to ED-based AMI care and mapping these barriers to targeted implementation strategies

After identifying the key barriers to ED-based AMI care, the CFIR-ERIC mapping tool was used to map these barriers to targeted implementation strategies [21]. The CFIR-ERIC mapping tool is a previously published instrument that provides recommendations from an independent panel of expert implementation scientists for effective implementation strategies for each CFIR barrier; [21] none of the members of our investigator team participated in the CFIR-ERIC expert panels. For any CFIR construct identified as a key barrier, only strategies with the strongest ERIC recommendations (“Level 1” strategies with endorsement by > 50% of experts) were included in the strategy mapping process. Figure 1 summarizes our entire analytic approach in this study.

### Ethics

This study received approval from ethical review committees at the Tanzania National Institute for Medical Research (NIMR/HQ/R.8a/Vol.IX/2436), Kilimanjaro Christian Medical Centre (Proposal 893), and Duke Health (Pro00090902). All participants provided written informed consent prior to enrollment. All the methods included in this study are in accordance with the declaration of Helsinki.

## Results

A total of 30 participants were enrolled in this study, including 10 patients, 10 providers, and 10 healthcare administrators (Table 1). Overall, 14 (47%) of participants were female and the median (IQR) age of all participants was 50 (30, 48) years. Patients had a range of educational backgrounds, and providers included physicians (*n* = 3), clinical officers (*n* = 3), and nurses (*n* = 4).

**Table 1:** Characteristics of patients, providers, and healthcare administrators participating in in-depth interviews (*N* = 30), northern Tanzania, 2021–2022

Patients (*N* = 10)		
Characteristic	n	(%)
Female sex	7	70%
Age, median (IQR), years	71 (51, 79)	
Any health insurance coverage	6	60%
Monthly household income, USD	21.8 (8.7, 519.8)	
Residence		
Urban	3	30%
Rural	7	70%
Highest education level achieved		
Some primary school	4	40%
Completed primary school	5	50%
Completed university	1	10%
Providers (*N* = 10)		
Female sex	2	20%
Age, median (IQR), years	29 (27, 30)	
Provider type		
Physician	3	30%
Clinical Officer	3	30%
Nurse	4	40%
Place of employment		
Referral hospital	6	60%
Community hospital	4	40%
Years of practice, median (IQR), years	3 (2, 5)	
Administrators (*N* = 10)		
Female sex	5	50%
Age, median (IQR), years	51 (47, 56)	
Place of employment		
Referral hospital	5	50%
Community hospital	5	50%
Administrator type		
Hospital Director	5	50%
Department head	2	20%
Charge nurse	3	30%

In the thematic analysis, 11 barriers to evidence-based AMI care in the ED were identified, corresponding to 9 CFIR constructs (Table 2). Key barriers included complexity and cost of AMI care, hospital culture, lack of resources, inadequate knowledge about AMI among providers and patients, need for quality improvement leaders, poor doctor-patient communication, and inefficient care systems. Most barriers were identified by multiple participants across all three stakeholder groups (Table 2).

**Table 2:** Key barriers to evidence-based AMI care in the Tanzanian ED and corresponding CFIR constructs

CFIR Domain	CFIR Construct	Key barrier	Stakeholders identifying this barrier (*N* = 10 for each group)	Illustrative quote
Innovation	Complexity	Providing timely and evidence-based AMI care would require substantial changes to current ED operations	5 Administrators 5 Providers 2 Patients	*I: “What would have to change to make immediate testing work for patients with chest pain or shortness of breath?”* *R: “We would need to have doctor in triage. Also we would need advice from you about other tests to do, because we are doing only blood pressure if patient comes in with difficulty breathing.”* (Hospital Director, community hospital)
Innovation	Cost	High-quality AMI care requires substantial hospital investments in expensive diagnostic equipment, treatments, staff, and other infrastructure.	8 Administrators 8 Providers 1 Patient	*“We have a plan to increase [diagnostic] equipment, but for that plan to be accomplished we need money. Therefore, if we get money in time, we can solve that. But also, if we get donors, we would be able to purchase the equipment.”* (Clinical Officer, community hospital)
Inner Setting	Culture	Some participants perceive a lack of urgency, motivation, or attention to detail among ED staff when caring for AMI patients.	5 Administrators 9 Providers 5 Patients	*“There is a sense of urgency in the cardiology practice, and I believe not all levels of health providers have that sense. They might suspect, but not everyone understands the essence of the problem, no one thinks of following patients closely. There are specialists who understand but speaking about health providers including nurses most people do not have the sense of urgency.”* (Physician, referral hospital)
Inner setting	Available resources	Many EDs do not have adequate staff, diagnostic equipment, and treatment capacity to care for AMI patients.	10 Administrators 10 Providers 6 Patients	*“I think one of our challenges is a lack of equipment for [AMI] treatment, and also lack of medication. And we lack enough competent staff to deal with heart attack problems.”* (Physician, referral hospital)
Inner setting	Access to knowledge and information	Many ED providers lack adequate training in the diagnosis and treatment of AMI.	10 Administrators 10 Providers 4 Patients	*I: “The doctors who were attending to you, did they say what the cause was?”* *R: “They said it was stress or smoking but I said I don’t smoke.”* (AMI Patient) *“We are not competent in caring for MI patients, but we try our best.”* (Hospital Director, community hospital)
Characteristics of individuals	Knowledge and beliefs about the innovation	Many patients lack basic understanding of AMI, both before and after their diagnosis.	10 Administrators 10 Providers 10 Patients	*I: “Can you tell me more about heart attack; do you know what that is?”* *R: “To be honest, I can’t explain what that is.”* (AMI Patient)
Process	Formally appointed internal implementation leaders	A formal leader is needed to supervise an AMI quality improvement initiative.	8 Administrators 8 Providers 0 Patients	*“I think when it comes to leadership, understanding is quite important. If you don’t know, you cannot do anything. Therefore as a team we should find a person with good communication skills who can take initiatives to motivate others and who has good organization skills, and they should be able to coordinate others in order to drive the team to succeed.“* *(Physician, referral hospital)*
Process	Champions	Multiple staff members are needed to encourage the care team to commit to improving AMI care.	3 Administrators 7 Providers 0 Patients	*“For this, I think everybody should be the leader in their position and an advocate to improve heart attack services.”* (Physician, referral hospital)
Process	Execution	EDs sometimes fail to provide high-quality AMI care, even when diagnostic and treatment capacity is available.	9 Administrators 7 Providers 6 Patients	*“Yes, at first I went to [first hospital] as normal and they advised that I reduce my workload. They asked what I was doing and they told me that I was overworking. That was not the case. Later I decided to go to a private hospital and the doctor said maybe according to your age, it might be that you are hitting menopause.”* (AMI Patient) *“There is a challenge of quality management because sometimes, some of the clinicians are not following the guidelines to initiate those AMI medicines. […] Someone came to me and said they have a challenge in diagnosing MI patients because they do not have ECG, so we provided an ECG machine, but tomorrow morning all the patients with difficulty breathing, all of them they did not get tested. It is not because they do not know how to use it, and if you ask them they don`t have specific reasons why not.”* (Department head, referral hospital)
	Execution	Providers sometimes do not communicate effectively with AMI patients or counsel them.	6 Administrators 4 Providers 9 Patients	*I: “Did the doctors explain what you are suffering from?”* *P: “They did not give me any explanation.”* (AMI patient) *“When I saw the doctor and explained what happened, he wrote down some medicines, and they gave me those, he didn’t say much about what could be wrong, I have seen that with a lot of doctors they don’t tell you what you are dealing with.”* (AMI patient)
	Execution	ED systems of care and patient flow processes are sometimes inefficient.	7 Administrators 6 Providers 1 Patient	*“Barriers are in the registration process. It takes a long time because of the large number of patients, more than 120 patients. So patients have to stand for too long, waiting for registration then after that they should go to triage, which also takes time because there are 2 nurses to attend 120 patients and instruments are few. After that there is a queue to see a doctor and doctors are few, so the queue is going very slowly. Then after seeing the doctor patients should go to the pharmacy or laboratory. Again, there is a queue. So patients become very tired due to this system. Another thing, sometimes errors may happen like skipping some codes of medicine or forgetting to fill some medication which takes a long time to resolve. So the challenges are many.”* (Charge nurse, referral hospital)

Table 3 presents the results of the CFIR-ERIC strategy mapping for the key barriers to ED-based MI care in Tanzania. Overall, 5 Level 1 strategies addressing 7 CFIR constructs were identified. Implementation strategies recommended for inclusion in the multicomponent intervention included: access new funding, identify and prepare champions, conduct educational meetings, develop educational materials, and distribute educational materials. There were no Level 1 implementation strategies for key barriers related to the CFIR constructs of complexity or execution.

**Table 3:** Results of CFIR-ERIC implementation mapping to identify key strategies to address barriers to ED-based AMI care in Tanzania

CFIR Construct	Key Barrier(s)	Implementation strategy
Complexity	Providing timely and evidence-based AMI care would require substantial changes to current ED operations	(No Level 1 strategy)
Cost	High-quality AMI care requires substantial hospital investments in expensive diagnostic equipment, treatments, staff, and other infrastructure.	Access new funding
Culture	Some participants perceive a lack of urgency, motivation, or attention to detail among ED staff when caring for AMI patients.	Identify and prepare champions
Available resources	Many EDs do not have adequate staff, diagnostic equipment, and treatment capacity to care for AMI patients.	Access new funding
Access to knowledge and information	Many ED providers lack adequate training in the diagnosis and treatment of AMI.	Conduct educational meetings; develop educational materials; distribute educational materials
Knowledge and beliefs about the innovation	Many patients lack basic understanding of AMI, both before and after their diagnosis.	Conduct educational meetings
Formally appointed internal implementation leaders	A formal leader is needed to supervise an AMI quality improvement initiative.	Identify and prepare champions
Champions	Multiple staff members are needed to encourage the care team to commit to improving AMI care.	Identify and prepare champions
Execution	EDs sometimes fail to provide high-quality AMI care, even when diagnostic and treatment capacity is available. Providers sometimes do not communicate effectively with AMI patients or counsel them. ED systems of care and patient flow processes are sometimes inefficient.	(No Level 1 strategy)

## Discussion

To our knowledge, this is the first study from SSA to systematically apply rigorous implementation science methods to identify barriers to evidence-based AMI care and map these barriers to implementation strategies. We identified 11 barriers to AMI care in the Tanzanian ED setting, which mapped to five evidence-based strategies: access new funding, identify and prepare champions, conduct educational meetings, develop educational materials, and distribute educational materials. Strategies were not identified using CFIR-ERIC matching to address barriers of complexity or execution of ED-based AMI care. These findings will inform the development of a contextually-tailored intervention to improve AMI care in Tanzania.

We identified barriers to care from four domains of CFIR. Many barriers were related to a lack of resources, inadequate education among both providers and patients, and local hospital culture. Multiple participants from all three stakeholder groups identified 9 barriers: complexity of AMI care, cost of providing high-quality AMI care, hospital staff culture, insufficient resources, inadequate provider training, lack of patient understanding, failure to execute high-quality care, poor provider-patient communication, and inefficient care systems. Two barriers (need for formally implementation leaders and champions) were only identified by providers and administrators, which is not surprising since patients are unlikely to have insight on the need for hospital-based implementation leaders.

The strategies identified by the CFIR-ERIC mapping approach, especially in combination, are likely to address these barriers and improve uptake of evidence-based care. Additional work is needed, however, to flesh out the operationalization of these strategies. For example, in our setting there is not an obvious path to accessing new funding for AMI care. Although new investments in ECG machines, laboratory-based troponin assays, hiring of additional ED and cardiology staff, and construction of a cardiac catheterization lab capable of performing percutaneous coronary intervention could all clearly contribute to improved AMI care in northern Tanzania, identifying sources of funding for these investments remains challenging. Moreover, expanding capacity would also require substantial additional staff training to ensure additional resources are used properly. Further creative engagement with stakeholders from the ministry of health, health insurance organizations, non-governmental organizations, research institutes, and industry partners are needed to develop robust and sustainable funding streams to expand capacity for AMI care in Tanzania. Our participants expressed strong support for further training for both patients and providers. Developing and distributing educational materials could clearly address barriers related to provider and patient knowledge, but further intensive work is needed to determine the content of these educational materials and how they should be delivered. In settings outside of SSA, varied methods have been used to educate physicians and patients about best practices in AMI care, including online modules, pamphlets, recurring lectures, and posters, among others [5, 28, 29]. In our setting, where health literacy among patients may be lower and providers may have certain preferences, some of these educational methods may not be feasible or acceptable. As in all implementation work, piloting of educational materials with the intended audience will be essential for maximizing effectiveness.

Importantly, we identified four key barriers to ED-based AMI care in Tanzania that did not have a Level 1 recommended ERIC strategy. These barriers included: timely evidence-based AMI care would require substantial changes to existing ED operations, the ED sometimes fails to provide evidence-based AMI care even when resources are available, inadequate communication between doctors and patients, and inefficient systems of ED care and flow. This illustrates some of the limitations of the CFIR-ERIC mapping tool, which have been previously noted by others [21, 30, 31]. Additional study is needed to determine effective strategies for these barriers which did not have strong recommendations in ERIC. Possible solutions include adjusting current ED flow in a minimally disruptive way to improve delivery of AMI care, using educational materials to enhance physician-patient communication, designation of a counselor to improve physician-patient communication, auditing of providers to give them feedback when they fail to provide evidence-based care, and re-designing of inefficient systems. The team that designs our multicomponent intervention to improve ED-based AMI care in northern Tanzania will need to further engage with providers and administrators to explore these barriers to care and identify locally-appropriate solutions to address them. Thus, we anticipate that the final intervention will incorporate additional strategies beyond the 5 strategies recommended by ERIC.

This study had several limitations. First our thematic analysis deliberately focused barriers to the ED phase of AMI care. Our participants identified additional barriers that would not be addressable by an ED-based intervention at a single hospital, such as health insurance policies and an Emergency Medical Transport system. Analyzing barriers and facilitators to inform interventions that address in pre-hospital, inpatient, and post-hospital phases of MI care will be the subject of a separate project. Secondly, in order to maximize local relevance, we only recruited informants from the Kilimanjaro Region of Tanzania. Therefore, our results may not be generalizable to other regions. Thirdly, as in all qualitative research, our data may have been subject to social desirability bias if participants shared perspectives that they believed would be most acceptable to our research team. In order to minimize this bias, our research assistants underwent rigorous training in best practices in conducting in-depth interviews to maximize participant comfort. Fourthly, as discussed above, although the CFIR-ERIC tool provides a rigorous approach for identifying effective strategies for implementing change, it was necessary to establish sub-constructs that more specifically identified barriers in this setting and several constructs did not lead to strong recommendations for strategies that could be used in our setting.

## Conclusions

In conclusion, using a rigorous implementation science approach, we identified multiple barriers to ED-based AMI care in Tanzania, which mapped onto five implementation strategies. Given the current paucity of published research addressing implementation of evidence-based AMI care in SSA, this study may provide a roadmap for teams working to improve AMI care elsewhere in SSA, where the barriers and contextual factors may have some similarities. Future work will build on these findings to develop and test a locally-tailored intervention to improve ED-based AMI care in our setting.

### Electronic supplementary material

Below is the link to the electronic supplementary material.

Supplementary Material 1

Supplementary Material 2

Supplementary Material 3

Supplementary Material 4

## Data Availability

The datasets generated and/or analyzed during the current study are not publicly available due to Tanzanian law, but are available from the corresponding author on reasonable request.

## Abbreviations

AMI: Acute myocardial infraction
CFIR: Consolidated Framework for Implementation Research
ERIC: Expert Recommendations for Implementing Change
ED: Emergency Department
SSA: Sub-Saharan Africa
ECG: Electrocardiogram
