# Supplementary material for: Improving acute myocardial infarction care in northern Tanzania: barrier identification and implementation strategy mapping
Source: BMC Health Serv Res. 2024 Mar 28;24:393. doi: 10.1186/s12913-024-10831-5 (PMC10979618; doi:10.1186/s12913-024-10831-5)
Supplement: Supplementary file 2 — Supplementary Material 2 [file 12913_2024_10831_MOESM2_ESM.docx]

**Barriers to MI care in Tanzania**

**INTERVIEW GUIDE FOR PATIENTS**

You are being asked to participate in this study because you have a history of having a heart problem called a heart attack or myocardial infarction. We would like to get your perspectives on ways to improve care of heart diseases like yours in Tanzania.

1. If you remember, can you tell me about what happened when you had your heart attack?
   1. What symptoms did you initially have? What did you think your symptoms were from?
   2. Did you see a doctor right away? Why or why not?
   3. When you did see a doctor, where did you go?
   4. What did the doctor or doctors tell you were the cause of your symptoms? What testing did they do? Did different doctors tell you different things about the cause of your disease?
   5. Did you have to see multiple doctors or go to multiple facilities? If so, why?
   6. What was the overall quality of your experience with the care you received for your heart attack? Was it good or bad or in between? Why?
2. Can you tell me more about a heart attack?
   1. How did your doctors explain this disease to you?
   2. What is your understanding of this disease? What causes it?
   3. What has this disease meant for you and your life?
3. Tell me more about the treatment you received for your heart attack.
   1. Did you receive any treatment immediately after you got the hospital? Did you receive any prescriptions? Do you remember what the medicines were or what they were for?
   2. Were you told by the doctor that you needed any follow-up appointments or additional testing? Did you follow-up? Why or why not?
   3. How long were you told to take the medicines for? Were you told you needed to refill the prescriptions? How were you told to do that?
   4. Did you take your medicines? For how long? Where did you get them from? Are you still taking those medicines? Why or why not?
4. Besides your heart problem, have you ever been diagnosed with other problems like high blood pressure, diabetes, or high cholesterol?
   1. Have you ever been tested for these?
   2. Have you ever been prescribed medications for these problems? Why or why not?
   3. Have you ever taken medicines for these problems? Why or why not? How long were you told to take them for?
   4. What follow-up have you been given for these problems?
   5. Do you feel these problems are well-controlled? Have you had any issues managing these problems?
5. What challenges have you faced in managing your heart disease?
   1. Did you have any challenges related to getting a diagnosis when you had a heart attack? Were there any tests that you were told you needed that you couldn’t get at some facilities?
   2. Did you have any challenges related to getting treatment for your heart attack? How about managing your heart attack now?
   3. Did you have any challenges related to cost?
   4. Did you have any challenges related to the health system or the referral system?
   5. Did you have any challenges related to your own health education and understanding of your disease?
   6. Have you had any challenges with follow-up appointments?
   7. Have you had any challenges with taking your medicines?
6. We are trying to understand how we can improve care for patients with heart disease. Do you have any suggestions for what we could do to improve the care of people with heart diseases like yours in the future?
   1. Would a patient education program be helpful? If so what do you think patients should be taught more about? Disease, medication, symptoms, exercise, diet?
   2. How do you think patients with heart diseases like yours should get more information about their disease and its management? Would a pamphlet be helpful? Or text messages? Or a phone call? Or one-one-counseling? Group meetings?
   3. Apart from patient education, what else could be done to improve the care of patients with heart diseases like yours in Tanzania?
7. Thank you for your time, is there anything else you want to say before we conclude?
