# Supplementary material for: Improving acute myocardial infarction care in northern Tanzania: barrier identification and implementation strategy mapping
Source: BMC Health Serv Res. 2024 Mar 28;24:393. doi: 10.1186/s12913-024-10831-5 (PMC10979618; doi:10.1186/s12913-024-10831-5)
Supplement: Supplementary file 3 — Supplementary Material 3 [file 12913_2024_10831_MOESM3_ESM.docx]

**Barriers to MI care in Tanzania**

**INTERVIEW GUIDE FOR ADMINISTRATORS**

You are being asked to participate in this study because you have an administrative role within a healthcare facility that cares for patients with MI. We are asking for your perspective on ways to improve MI care at facilities like yours.

1. What do you think are the primary barriers to high-quality MI care at your facility?
   1. Are there barriers related to equipment?
   2. Are there barriers related to provider training/education?
   3. Are there barriers related to cost?
   4. Are there barriers related to the system?
   5. Are there any barriers related to the patients?
   6. Are there barriers related to culture?
   7. Are there any other barriers that we haven’t talked about yet?
   8. Here are some recommendations for high-quality MI care from the WHO and other countries. Do you think this are achievable at your facility? Are there any barriers to accomplishing these at your facility?
      1. Immediate testing with ECG for any patient presenting with possible MI symptoms
      2. Immediate aspirin administration if concern for MI
      3. Troponin/cardiac biomarker testing if concern for MI
      4. Heparin treatment if confirmed MI
      5. By the time of hospital discharge, initiation of beta blocker, ACE inhibitor or ARB, statin, and antiplatelet therapy like aspirin. Indefinite treatment with these therapies unless complications or contraindications.
      6. Counseling on smoking cessation, importance of weight loss if overweight, healthy diet including reducing salt and fat, exercise.
2. What do you think could be done to improve:
   1. Diagnosis of MI?
   2. Reducing missed cases of MI?
   3. Treatment of MI in the emergency department and hospital?
   4. The discharge process of patients with MI?
   5. Appropriate use of evidence-based medications after hospital discharge for patients with MI?
   6. Medication adherence among patients with MI?
   7. Patient education and counseling?
   8. Follow-up care after hospital discharge for patients with MI?
   9. Management of comorbidities such as hypertension, diabetes, high cholesterol among patients with MI?
3. Here are examples of some things that have been done in other countries to try to improve MI care. Do you think these would work at your facility? What would have to change to make this work at your facility?
   1. Nurse-driven triage protocol to automatically obtain EKG and troponin immediately for any adult presenting with chest pain or shortness of breath.
   2. Checklists to remind providers about emergency and inpatient care for patients with MI
   3. Discharge checklists to make sure all appropriate medications had been written.
   4. Patient educational materials about MI
   5. Electronic medical system reminders to give aspirin and prescribe secondary preventative medications
   6. Auditing care and giving feedback to providers (example: telling providers when they forget to prescribe aspirin)
   7. Educational sessions for nurses and doctors
   8. Specialized follow-up clinic
4. At your specific healthcare facility, what are unique factors that need to be considered?
   1. Are there any important factors related to the personnel or staff?
      1. Who (job title) at your facility would be the best person to lead efforts to improve MI care? Why?
   2. Are there any important factors related to culture?
   3. Are there any important factors related to resources?
   4. Are there any important factors related to the local community?
   5. Are there any important factors related to care processes?
   6. Are there any other important factors that would need be considered before developing an intervention to improve MI care at your facility?
5. We are trying to understand how we can improve care for patients with MI. Do you have other any suggestions for what we could do to improve the care of people with MI in Tanzania?
6. Thank you for your time, is there anything else you want to say before we conclude?
